# Correlating Intracranial Pressure Following Decompressive Craniectomy With Neurological Outcomes in Severe Traumatic Brain Injury Patients: A Prospective Observational Study

**DOI:** 10.7759/cureus.40119

**Published:** 2023-06-08

**Authors:** Abhijit Ravindra Chandankhede, Snehal D Thombre, Dhanwantari Shukla

**Affiliations:** 1 Neurosurgery, Shree Siddheshwar Multispeciality Hospital, Dhule, IND; 2 Anesthesiology, Shree Siddheshwar Multispeciality Hospital, Dhule, IND; 3 Neurosurgery, The Neurocity Hospital, Varanasi, IND

**Keywords:** correlation of icp with neurological outcome, neurological outcome, decompressive craniectomy, severe traumatic brain injury, intracranial pressure monitoring

## Abstract

Introduction

Decompressive craniectomies have been performed in settings with raised intracranial pressure (ICP) after severe traumatic brain injury (TBI). A decompressive craniectomy (DC) is an important salvage procedure for intracranial hypertension. The changes in the intracranial microenvironment after a primary DC are significant in terms of the neurological outcome in the postoperative period.

Materials and methods

The study comprised 68 patients with severe TBIs who were undergoing primary DC; of these, 59% were male. Recorded data include demographic profiles, clinical features, and cranial computed tomography (CT) scans. All patients underwent a primary unilateral DC with augmentation duraplasty. Intracranial pressure was recorded in the first 24 hours at regular intervals, and the outcome was recorded using the Extended Glasgow Outcome Scale (GOS-E) at two-week and two-month intervals.

Results

Road traffic accidents (RTAs) are the most common cause of severe TBIs. Imaging studies and intraoperative findings suggest that acute subdural hematomas (SDHs) are the most common pathology leading to high ICP in the postoperative period. Mortality was strongly statistically associated with high ICP values postoperatively at all intervals. The average ICP for the patients who died was 11.871 mmHg higher than the patients who survived (p=0.0009). The Glasgow Coma Scale (GCS) at the time of admission is positively correlated with the neurological outcome at two weeks and two months, with a Pearson correlation coefficient of 0.4190 and 0.4235, respectively. There is a strong negative correlation between ICP in the postoperative period and the neurological outcome at two weeks and two months (Pearson correlation coefficients are −0.828 and −0.841, respectively).

Conclusion

The results indicate that RTAs are the most common cause of severe TBIs, and acute SDHs are the most common pathology leading to high ICP after the surgery. ICP values in the postoperative period have a strong negative correlation with survival and neurological outcome. Preoperative GCS and postoperative ICP monitoring are important methods of prognostication and planning further management.

## Introduction

Traumatic injury is a poorly recognized global public health concern, accounting for a significant cause of morbidity and mortality in nearly every nation and population. Low- and middle-income nations account for 89% of all trauma-related deaths [1,2]. Traumatic brain injury (TBI) is defined as an alteration in brain function or other evidence of brain pathology caused by an external force because it occurs in all regions and affects people of all ages and income groups [3,4]. A TBI presents as one of the following: a decreased level of consciousness, amnesia, objective neurologic or neuropsychological abnormality, skull fracture(s), diagnosed intracranial lesion(s), or head injury listed as a cause of death on a death certificate [5,6]. A TBI disrupts the intracranial environment with multiple disturbances in the form of intracranial hypertension, altered circulation, and disturbed healing. Subdural hematomas (SDHs) and contusions in more than 50% of severe TBI cases are associated with mass lesions. The vicious cycle of mechanical compression, edoema, hypoxia, and ischemia leads to rapidly deteriorating secondary injuries that require urgent surgical intervention. Intracranial hypertension is the major cause of secondary brain injury, leading to morbidity and death. Thus, treatment protocols are guided to control ICP in patients with TBI [7].

Clinical evidence to allow for an unbiased treatment choice for severe TBI is insufficient [8]; however, notable clinical trials include RESCUE-ICP and RESCUE-ASDH [9,10]. Although decompressive craniectomy (DC) has been advocated as one strategy for managing intracranial pressure (ICP) [11], ICP monitoring has generally been approved in evidence-based guidelines for patients with severe TBI [12,13]. ICP waveform analysis and understanding have improved tremendously over the last few decades, but there is still a lack of "class I" evidence that supports the superiority of treatment based on ICP monitoring over treatment guided by neurological testing and serial CT imaging in improving short-term or long-term recovery in the general population of patients with a severe TBI [11]. One of the knowledge gaps is the role of ICP monitoring in TBI patients after a primary DC. In this context, "primary" refers to a DC during the evacuation of an intracranial lesion in the acute phase. Monitoring ICP in the initial decompression area may provide pertinent diagnostic information leading to more appropriate postoperative medication that reduces ICP or a well-timed treatment [14]. The purpose of this study is to evaluate the correlation of postoperative ICP monitoring during an early DC with mortality and neurological outcome as well as to guide therapy in patients who undergo an early DC for a severe TBI.

## Materials and methods

This study included patients aged between 18 and 70 who had TBIs and/or traumatic mass lesions and underwent a DC. The study was conducted at a tertiary care centre in India from March 2017 to May 2018. Patients under 18 and over 70 years of age were excluded, as well as those with severe extra-cranial lesions (such as cardiovascular, pulmonary, or abdominal injuries), systemic comorbidities (like ischemic heart disease, chronic kidney disease, and uncontrolled diabetes), a brain death diagnosis upon admission, and penetrating brain injuries.

Data collection included age, sex, Glasgow Coma Scale (GCS), pupil size, and imaging studies (such as non-contrast computed tomography (CT) and/or magnetic resonance imaging) to confirm the type of injury, hematoma, side of the lesion, type of lesion, side of surgery, type of surgery, and intraoperative and immediate postoperative complications. ICP measurement was conducted using the intraventricular ICP transducer monitoring device (Codman ICP Express), with measurements taken at regular intervals after surgery (immediate postoperative, six hours, 12 hours, and 24 hours in the postoperative period). The Glasgow Outcome Scale-Extended (GOS-E) was assessed at two weeks and two months after surgery (Table 1).

**Table 1:** Glasgow Outcome Scale - extended score [15]

Score	Label	Interpretation
1	Dead	Dead
2	Vegetative state	Absence of awareness of self and environment
3	Lower severe disability	Needs full assistance in daily activity of living
4	Upper severe disability	Needs partial assistance in the activities of daily living
5	Lower moderate disabilities	Independent but cannot resume work school or all previous activities
6	Upper moderate disabilities	Some disability exists but can partially resume work or previous activities
7	Lower good recovery	Minor physical and mental disabilities that affect daily life
8	Upper good recovery	Full recovery or minor deficits that do not affect the daily activities

For the demographic profile, descriptive statistics were used to summarise the age distribution of the participants, including the mean age, standard deviation, minimum age, and maximum age. The proportions of males and females were also calculated. A statistical test, specifically a chi-square test, was conducted to determine the statistical significance of the difference in the number of male and female patients across all age groups. In the overall outcome analysis, the GOS-E was used to measure patients' outcomes. The range of GOS-E scores was reported, and the number of patients who died at the end of two weeks was provided. It is mentioned that statistical analysis was conducted to determine the association between patient characteristics (age, gender) and treatment outcomes (death), as well as the association between mortality and postoperative ICP values.

Collected data were statistically analysed using SPSS 24 (IBM Corp., released 2016, IBM SPSS Statistics for Windows, Version 24.0, Armonk, NY: IBM Corp.) and Real Statistics Resource Pack software (Release 6.2), copyright (2013-2019), Charles Zaiontz (www.real-statistics.com).

This study is approved by the institutional review board and ethics/scientific committee (approval no. ECR/246/tnst/OR/2013/RR-2016/PR-N-37).

## Results

Sixty-eight participants with TBIs were studied in terms of clinical aspects and outcomes after a DC. The collected data were analyzed following the statistical procedure described in the methodology, and the interpretation of the results is presented in the following sections.

In this study, 68 subjects of varying ages were enrolled. Among them, 34 subjects (51.5%) were aged between 30 and 50, 28 subjects (41.2%) were over 50, and six subjects (7.4%) were under 30. The average age of the patients was 47.23 years, with a standard deviation of 13 years. Out of the 68 patients, 40 were male (58.8%) and 28 were female (41.2%). The proportion of male patients was statistically significant (p=0.02103642) across all age groups. The age range of the patients was from 23 to 68 years, with an average age of 47.235±12.498.

Cause of injury

Road traffic accidents (RTAs) caused most of the head injuries in patients that were admitted to the hospital (58 out of 68 enrolled subjects; 85.29%). The remaining 10 patients (14.70%) suffered a head injury due to a domestic fall. However, there was no statistically significant difference in the frequency between males and females regarding a fall or an RTA as a cause of a head injury (p=0.9203).

Overall outcome analysis

The patients’ outcomes were defined using the GOS-E (Table 1), which ranged from 1 to 8. GOS-E 1 defines death at the given outcome assessment interval. Eight patients died at the end of two weeks (11.76%), the details of which are shown in Table 2. The mortality rate did not change at the end of two months, i.e., no new patients died at the end of two months. The GOS-E ranged from 1 to 7 for all patients at the end of two weeks and from 1 to 8 at the end of two months. Neither patient age nor gender seemed statistically significant in terms of death as the treatment outcome; however, mortality was strongly statistically associated with high ICP values postoperatively at all intervals. The average ICP for the patients who died was 11.871 mmHg higher than the patients who survived (p=0.0009; Table 2).

**Table 2:** Mortality profile

Mortality - 8 patients			
Age	Min	42	p=0.0507
	Max	65	
	Mean	48.5	
	Standard deviation	10.212	
Sex	Males	4	
	Females	4	
Laterality	Left	8	
	Right	0	
ICP	Min	27.75	p=0.0009 95%CI for mean=28.8445 to 39.0155
	Max	42.5	
	Standard deviation	6.084	

The majority of patients had a GOS-E score of 6 at the end of two weeks (38.23%), and the majority of patients had a GOS-E score of 7 at the end of two months (23.52%). The mean GOS-E score at the end of two weeks was 4.58 with a standard deviation of 1.78, and the mean GOS-E score at the end of two months was 5.41 with a standard deviation of 2.267. There was a statistically significant GOS-E improvement in the outcome from two weeks to two months with p=0.00005024 and a chi-square value of 31.501 (Table 3).

**Table 3:** Individual GOS-E score distribution GOS-E: Glasgow Outcome Scale-Extended

Outcome	GOSE At 2 weeks		At 2 months	
	Frequency	%	Frequency	%
1	8	11.76	8	11.76
2	2	2.94	2	2.94
3	6	8.82	6	8.82
4	12	17.64	2	2.94
5	10	14.7	10	14.7
6	26	38.23	12	17.64
7	4	5.88	16	23.52
8	0	0	12	17.64
Total	68	100	68	100
Average	4.58		5.41	
Standard deviation	1.780787		2.267455	

**Figure 1:**
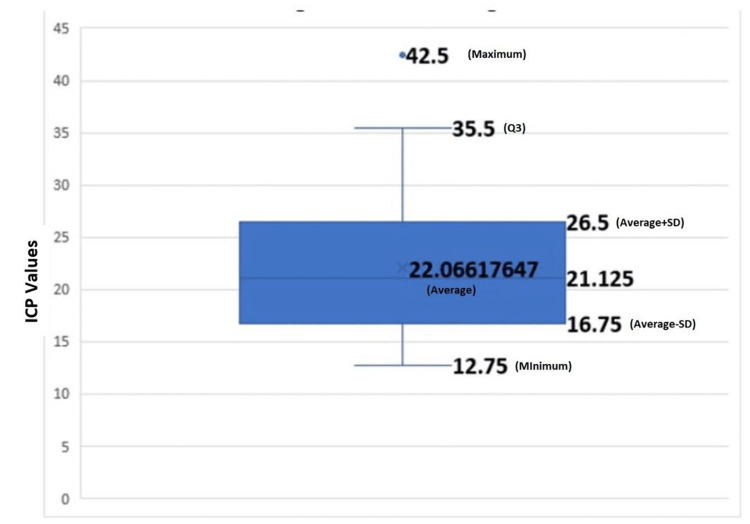
Average intracranial pressure

The average ICP values across all intervals provide a detailed analysis of the range of ICP in the first 24 hours after surgery. The minimum average ICP was 12.75 mmHg, and the maximum was 42.5 mmHg. The mean average ICP was 22.066 with a standard deviation of ±7.11 mmHg (Figure 1).

Association between average ICP and outcome at two-week and two-month intervals 

After two weeks, when the association between the average ICP and outcome parameters was established, a lower ICP was associated with a better outcome in terms of the GOS-E score (Fisher’s exact test, p<0.001; Table 4).

**Table 4:** Association of average intracranial pressure with outcome at two weeks

		ICP average (mmHg)		Total
Outcome at 2 weeks GOSE		>20	<20	
	1 to 4	26	2	28
	5 to 8	12	28	40
Total (p-value <0.001)		38	30	68

The above trend was reinforced by the association between the average ICP and the outcome in terms of GOS-E after two months. An average ICP of more than 20 mmHg was associated with poorer GOS-E scores of 1-4, and an average ICP below 20 mmHg was associated with better outcome scores of 5-8 on GOS-E (Fisher’s exact test, p=0.00000264; Table 5).

**Table 5:** Association of average intracranial pressure with outcome at two months

		ICP average (mmHg)		Total
Outcome at 2 months (GOSE)		>20	<20	
	1 to 4	18	0	18
		47.4%	0	26.5%
	5 to 8	20	30	50
		52.6%	100%	73.5%
Total (p-value = 0.00000264)		38	30	68
		100%	100%	100%

**Figure 2:**
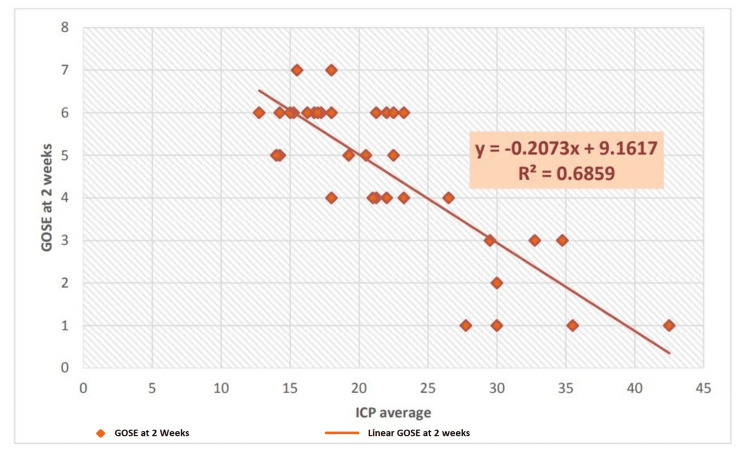
Correlation of average intracranial pressure with GOS-E at two weeks GOS-E: Glasgow Outcome Scale-Extended

Correlation between the average ICP and GOS-E at two weeks postoperatively. Pearson’s correlation demonstrated that the average ICP values were correlated with the GOS-E scores over the two-week postoperative period. A correlation value of −0.832 means that if ICP is lower, the outcome scores (5-8) are better (p<0.001), and if ICP values are high, the GOS-E outcome scores are low (1-4). A similar correlation is ascertained with the calculation of R²=0.6859 for the average ICP and GOS-E scores at two weeks. A graphical representation of this is shown in Figure 2.

**Figure 3:**
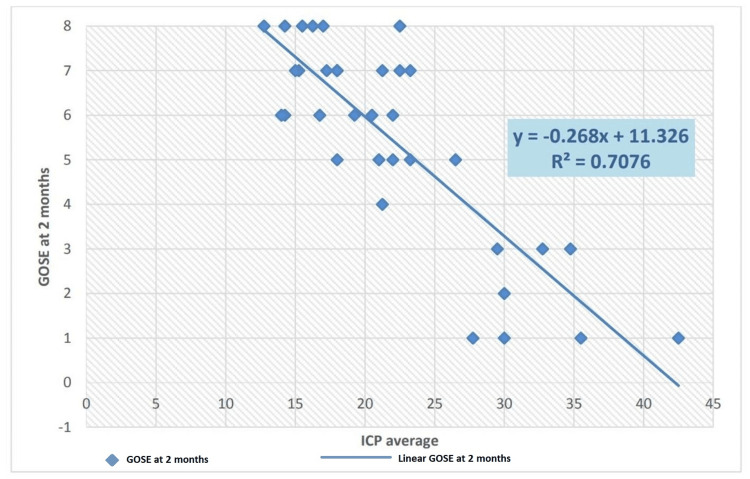
Correlation of average intracranial pressure with GOS-E at two weeks GOS-E: Glasgow Outcome Scale-Extended

Correlation between the average ICP and GOS-E at two months postoperatively. Pearson’s correlation shows that the average ICP values are correlated with the GOS-E scores during the two-month postoperative period. A correlation value of −0.841 means that if ICP is lower, outcome scores (5-8) are better (p<0.001), and if ICP values are high, GOS-E outcome scores are low (1-4). A similar correlation is ascertained with the calculation of R²=0.7076 for the average ICP and GOS-E scores at two months. A graphical representation of this is shown in Figure 3.

**Figure 4:**
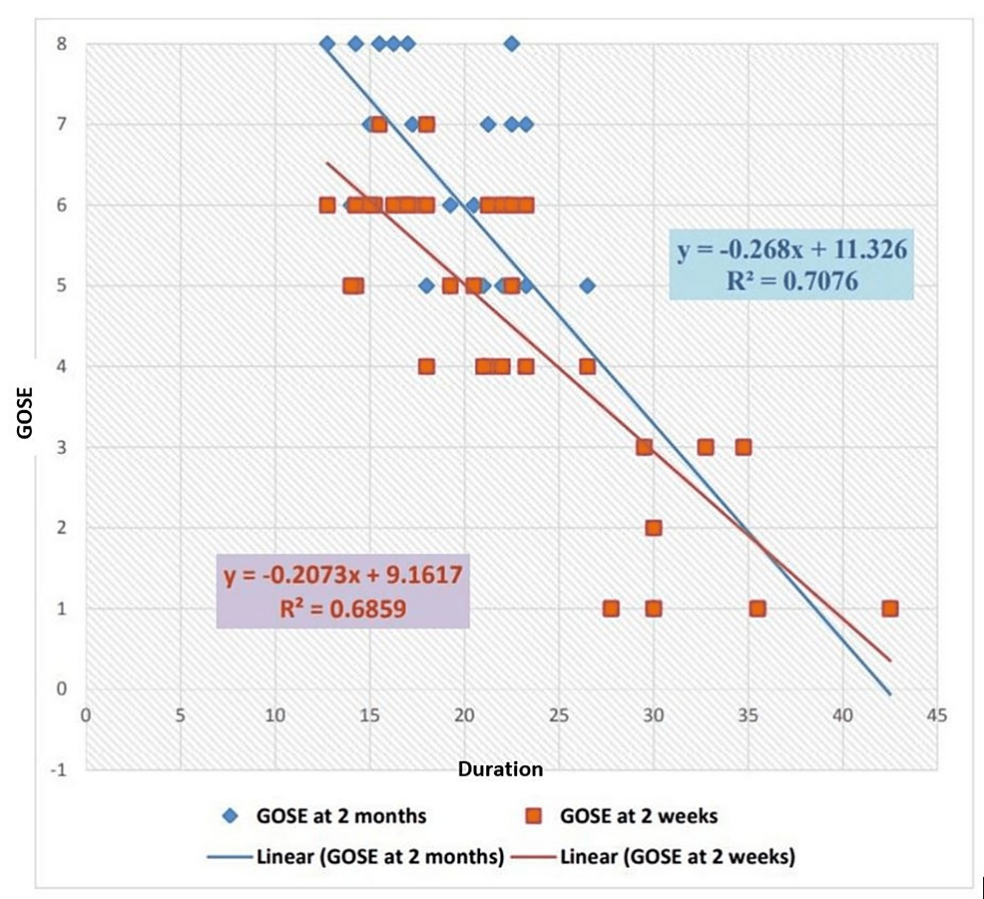
Combined trend of average intracranial pressure and outcome correlation at two weeks and two months

Figure 4 combines the graphical trends of average ICP with GOS-E scores at two weeks and two months.

ICP monitoring produced complications that included ventriculitis, meningitis, and long-term cerebrospinal fluid leaks. However, respiratory tract infections, urinary tract infections, and other intensive care unit-related complications were recognized and managed accordingly.

## Discussion

This observational study investigated clinical aspects and outcomes in TBI patients after a DC. The findings include information on demographics, clinical manifestations, and outcomes.

Age and gender distribution across various brain injury groups

This study included 68 patients who underwent primary DC for a TBI. Eight of these patients died during the two-week interval. The study sample had a male preponderance (59%), and 41% of the sample was female. Previous studies have included a similar male preponderance of 82.05% [16] and 61.8% [17]. TBI in males is attributed to the fact that male subjects are more exposed to RTAs. Males are generally more exposed to road traffic accidents due to several factors. These factors include differences in driving behaviour, risk-taking tendencies, and higher engagement in activities such as driving motorcycles or heavy vehicles. Additionally, cultural and societal norms may contribute to higher rates of male involvement in road traffic accidents and, thus, consequential head injuries. We observed that most of the patients in our study were aged between 30 and 50 (51.5%), and the second biggest group was those aged over 50 (41.2%). Only 7.4% of patients were under 30 years old. The mean age was 47.23 with a standard deviation of 13 (47.23±13). In a study [16], the patients were 44±17.6 years (18-80 years old), while in a study by Picetti et al. [17], the age recorded was 51.3±21.0 years. Similarly, Huang and Ou [18] had a mean age of 46.2±20.4 (1-88 years old), and in the study by Khalili et al. [19], the mean age of the patients undergoing a DC was 34.8±15.5 years (15-85 years old).

Cause of injury

In this study, RTAs caused a TBI in 58 of the patients, and the remaining 10 patients suffered a head injury due to a domestic fall (14.70%). No head injuries were caused by a penetrating trauma or missile. The data show that 8.82% of males suffered a head injury due to a fall, but 50% were caused by an RTA. The average age for a fall as a cause of a head injury was 49.8±11.34 years and 46.79±12.81 years for an RTA. The average age of patients involved in an RTA was less than that of those who fell. The minimum age for a fall was 35 and the maximum was 67, while the minimum age for an RTA was 23 and the maximum was 68. However, there was no statistically significant difference in the frequency between males and females regarding a fall or an RTA as a cause of head injury (p=0.92034433). When compared with previously published studies, the findings are quite similar, except for a study in 2019 that found that RTAs caused fewer head injuries [20].

GCS at the time of admission

The minimum GCS at the time of admission was 3, and the maximum was 8, with a mean of 5.529 and a standard deviation of 1.3211. When divided into two groups depending on the GCS, the GSC 3-5 group consisted of 38 patients (55.88%) and the GCS 6-8 group consisted of 30 patients (44.11%). These findings are comparable to those of previously published studies. In a study [21], the GSC 3-5 group comprised 57.74% of total patients, and the GCS 6-8 group comprised 42.25%. The mean GCS in the study by Picetti et al. [17] was 4, and in another study [22], it was 5.5±1.53. The mean GCS in our study was 5.529±1.32. Estimating the individual GCS scores for all patients in our study shows that 32.35% of patients were admitted to the emergency room with a GCS of 5, while 20.58% of patients were admitted with a GCS of 4. The percentage of patients with individual GCS scores is comparable to the recently published study by Zhao et al. [21].

Overall outcome analysis

Patient outcome was defined using the GOS-E, which ranged from 1 to 8 in our study. Eight patients had a GOS-E of 1, which means that they died at the end of the outcome assessment interval at the end of two weeks (11.76%). The age or gender of patients did not seem to be statistically significant in terms of mortality rate after treatment, but there was a strong association between high postoperative ICP values and mortality. The average ICP for the patients who died was 11.871 mmHg higher than the patients who survived (p=0.0009). The mortality rate did not change at the end of two months, i.e., no new patients died during this time. The GOS-E ranged from 1 to 7 for all patients at the end of two weeks and from 1 to 8 at the end of two months. The majority of patients had a GOS-E score of 6 at the end of two weeks (38.23%), and the majority of patients had a GOS-E score of 7 at the end of two months (23.52%). The mean GOS-E score at the end of two weeks was 4.58 with a standard deviation of 1.78, and the mean GOSE score at the end of two months was 5.41 with a standard deviation of 2.267. The mean GOS-E score reported by the study by Khalili et al. [19] at the end of two months was 3.48±2.19.

There was a statistically significant improvement in the outcome from two weeks to two months regarding the GOS-E score, with p=0.00005024 and a chi-square value of 31.501. An unfavourable outcome (GOS-E 1-4) is reported as 68% in the study by Zhao et al. [21] at the end of six months. Huang and Ou [18] reported favourable outcome scores in 41.2% of patients and unfavourable scores in 58.8% of patients at the time of discharge and follow-up. Similarly, Khalili et al. [19] reported 45.8% favourable outcome parameters and 54.2% unfavourable outcomes. Outcomes were compared by Chang et al. [20] in terms of GOS-E scores at six months and average ICP values in the postoperative period, with the result that favourable outcomes were obtained in 42.9% of patients in the ICP-monitored group. When transcranial Doppler monitoring and ICP monitoring were combined, this number increased to around 70%, which is explained by the early detection of intracranial hypertension and timely measures to control it. Similar findings were reported in a study by Picetti et al. [17], who found that unfavourable neurological outcomes were associated with greater mean ICP and lower CPP min measurements. A statistically significant relationship was found between the mean ICP after DC and GOS (p<0.04) and between the CPP min after DC and GOS (p<0.04). This study supports the evidence that postoperative episodes of intracranial hypertension have a negative impact on overall patient outcomes and demonstrates that higher postoperative ICP values are associated with unfavourable neurological outcomes (for two-week and two-month postoperative intervals, p<0.001). In our study, Pearson’s correlation shows that average ICP values are correlated with GOS-E scores during the two-week postoperative period. A correlation value of −0.828 means that if the ICP is lower, the outcome scores (5-8) are better (p<0.001), and if the ICP values are high, the GOS-E outcome scores are low (1-4). A similar correlation is ascertained with the calculation of R²=0.6859 for average ICP and GOS-E scores at two weeks. ICP and GOS-E scores at two months reveal a similar correlation, with Pearson’s correlation coefficient of −0.841 suggesting a strong negative correlation between ICP values and neurological outcomes as determined by the GOS-E score. This finding is substantiated by the calculation of robust regression for the same set of data (R^2^=0.7076). A similar robust regression correlation is presented by a study [22], which found a strong negative correlation between ICP values in patients undergoing a DC for severe TBI and neurological outcomes after a follow-up interval.

Limitation of the study

Our sample size only contained 68 patients. The patients satisfying the selection crieteria during the study period were added in the study. More patients are needed to provide a better overview of the statistical analysis. Furthermore, this was not a randomized study, and the follow-up period was only two months. The placement of intraventricular ICP monitor probes does not remove the chances of infection, so more non-invasive methods of proper ICP monitoring are needed for further infection-free monitoring. Research is also warranted for making ICP monitoring probes cost-effective and available so that more scientific-based management of patients can be carried out. we are looking foreward for the furether efforts of the researchers in this feild for better understanding of the concept.

## Conclusions

Traumatic brain injury is a rising global trend. Preoperative evaluation and management are needed to decrease the mortality and morbidity arising from this condition. Young adults suffer most from head injuries due to RTAs, in which SDHs and contusions warrant a DC. Preoperative GCS and postoperative ICP monitoring are vital for prognostication and planning further management. This study suggests that ICP values in the postoperative period strongly correlate negatively with survival and neurological outcomes. The data should be confirmed in future randomized controlled studies with larger sample sizes and longer follow-ups.
